# Zirconia Implants Indicated Better Stability After Exposure to Radioiodine-131 Therapy Used for Differentiated Thyroid Cancer

**DOI:** 10.3390/cancers17040678

**Published:** 2025-02-17

**Authors:** Alexandru Mester, Doina Piciu, Marioara Moldovan, Codruta Sarosi, Stanca Cuc, Ioan Petean, Cristina Moisescu-Pop, Andra Piciu, Florin Onisor, Simion Bran

**Affiliations:** 1 Department of Oral Health, University of Medicine and Pharmacy “Iuliu Hatieganu”, 400012 Cluj-Napoca, Romania; mester.alexandru@umfcluj.ro; 2 Doctoral School, University of Medicine and Pharmacy “Iuliu Hatieganu”, 400012 Cluj-Napoca, Romania; doina.piciu@umfcluj.ro; 3 Institute of Chemistry “Raluca Ripan”, University Babes-Bolyai, 400294 Cluj-Napoca, Romania; marioara.moldovan@ubbcluj.ro (M.M.); liana.sarosi@ubbcluj.ro (C.S.); stanca.boboia@ubbcluj.ro (S.C.); 4 Faculty of Chemistry and Chemical Engineering, Babes-Bolyai University, 400084 Cluj-Napoca, Romania; ioan.petean@ubbcluj.ro; 5 Department of Endocrine Tumors and Nuclear Medicine, Institute of Oncology “Ion Chiricuta”, 400015 Cluj-Napoca, Romania; mg.cristina@iocn.ro; 6 Department of Medical Oncology, University of Medicine and Pharmacy “Iuliu Hatieganu”, 400012 Cluj-Napoca, Romania; 7 Department of Maxillofacial Surgery and Implantology, University of Medicine and Pharmacy “Iuliu Hatieganu”, 400012 Cluj-Napoca, Romania; florin.onisor@umfcluj.ro (F.O.); dr_brans@umfcluj.ro (S.B.)

**Keywords:** radioiodine-131, I-131, dental implant, osseointegration, differentiated thyroid cancer

## Abstract

**Background:** Advancements in therapeutic approaches and standard medical interventions have significantly improved the prognosis of patients with differentiated thyroid cancer. However, uncertainties remain regarding the optimal timing and protocols for dental implant placement in patients undergoing radioiodine-131 (I-131) therapy. Debates continue about the potential effects of radiation on osseointegration dynamics and implant viability. This in vitro study assessed the impact of radiodiodine-131 (I-131) used for differentiated thyroid cancer on the structure of zirconia and titanium implants. **Methods:** A total of 60 implants were utilized, with distribution into two cohorts: titanium implants (Ti, *n* = 30) and zirconia implants (Zr, *n* = 30). Subsequently, the Ti and Zr implants were immersed in I-131 solution and retrieved at specified time intervals: 0, 6, 12, 24, 48 h, and 8 days post irradiation. The analyses used to characterize the structure of the implants were radioactivity, scanning electron microscopy, atomic force microscopy, roughness, and Vickers hardness assessment. **Results:** The findings indicate that the zirconia implants exhibited minimal ultra-structural topographic changes after irradiation. Notable topographical changes and debris deposition on zirconia surfaces became evident after 24 h, with cumulative effects observed after 192 h. The titanium implants, on the other hand, showed surface alterations beginning at 12 h of exposure. Significant changes, including erosive patterns and substantial debris deposits, occurred after 48 and 192 h, leading to increased surface roughness by 24 h. Implants exposed for 12 and 24 h formed a statistically significant group, indicating the onset of surface alteration accumulation. The erosion debris confirmed the surface alterations induced by radioiodine-131 exposure. **Conclusions:** Overall, the Zr implants demonstrated greater stability compared to the Ti implants following radioiodine-131 exposure.

## Simple Summary

This study looks at how radioiodine-131 (I-131) therapy affects titanium and zirconia dental implants. Zirconia implants showed minimal changes, even after long exposure, while titanium implants experienced significant surface damage after just 12 hours. Overall, zirconia implants were more stable and resistant to radiation, making them a better option for patients receiving I-131 therapy.

## 1. Introduction

The prognosis of patients diagnosed with differentiated thyroid cancer (DTC) has notably improved owing to advancements in therapeutic approaches and standard medical interventions [1,2]. Nonetheless, as patients with DTC experience prolonged life expectancies, the effective management of both the immediate and late side effect late ramifications of radioiodine-131 (I-131) therapy, the standard adjuvant therapy used in most of advanced DTC, has become increasingly imperative, aiming to enhance patients’ quality of life [3].

Among the multitude of factors influencing the well-being of these individuals, the management of oral complications arising from I-131 therapy, encompassing difficulties in mastication, articulation, deglutition, tooth integrity, and concomitant alterations in facial esthetics, emerges as pivotal [4,5,6]. Despite inherent challenges, oral rehabilitation utilizing either removable or fixed prostheses remains a prevalent strategy to ameliorate such adverse effects and sustain optimal oral functionality [6].

For irradiated oncological patients encountering impediments precluding the use of conventional dentures, implant-supported prostheses represent a pragmatic alternative, particularly in the presence of radiation-induced sequelae such as xerostomia, fragile mucosa, osteoradionecrosis, or anatomical distortions [6,7,8]. However, uncertainties persist regarding the optimal timing and protocols for dental implant placement in individuals subjected to I-131 therapy, with ongoing debates surrounding the potential interference of radiation with osseointegration dynamics and implant viability [2,6,9]. Notably, the absence of consensus papers concerning the radiation threshold levels compatible with preserved implant survival rates underscores the need for comprehensive investigations into the underlying mechanisms governing the interaction between I-131 exposure and osseointegration processes [2,6,10,11,12].

In light of the aforementioned uncertainties, our in vitro investigation aims to compare the impact of varying time intervals of I-131 exposure on the structural integrity of zirconia and titanium implants, aiming to provide insights into the optimal timing for initiating oral rehabilitation with dental implants in patients undergoing I-131 therapy. By navigating the intricate interplay between I-131 therapy and oral rehabilitation, clinicians can tailor interventions to mitigate adverse effects effectively, thereby optimizing the overall well-being and quality of life for individuals navigating the complexities of DTC management.

## 2. Materials and Methods

### 2.1. Sample Preparation for I-131 Irradiation

A total of 60 implants were utilized, with distribution into two cohorts: titanium implants (Ti, *n* = 30) and zirconia implants (Zr, *n* = 30). The preparation of the I-131 solution followed a previously validated protocol, documented in previous in vitro studies [13,14]. Each implant was individually immersed in an equal volume of I-131 solution and artificial saliva. Subsequently, the Ti and Zr implants were immersed in the prepared I-131 solution and retrieved at specified time intervals: 6, 12, 24, 48 h, and 8 days post irradiation. Control implants (Ti, *n* = 5; Zr, *n* = 5) were immersed in artificial saliva without I-131 exposure, and their structural properties were monitored over the same time intervals. These control implants were used for comparative analysis to distinguish the effects of I-131 exposure from any potential changes due to saliva immersion alone. Strict protocols were followed to prevent cross-contamination during sample preparation. The implants designated for exposure were immersed in separate containers with individually prepared I-131 solutions. Control implants were handled in a separate environment and confirmed to have no detectable activity. The radioactivity of each implant was quantified through serial measurements performed five times, and the mean values expressed in microcurie (µCi) were calculated using the Curiementor (Curiementor 3, Freiburg, Germany).

### 2.2. Scanning Electron Microscopy Assessment

The microstructural analysis of the irradiated samples was performed utilizing scanning electron microscopy (SEM). The SEM examination was carried out in low-vacuum mode with an acceleration voltage of 20 kV using an Inspect™ SEM microscope (FEI Company, Hillsboro, OR, USA).

### 2.3. Atomic Force Microscopy Assessment

The ultra-structural properties of the irradiated sample surfaces were investigated using atomic force microscopy (AFM) on a JEOL JSPM 4210 Scanning Probe Microscope (JEOL Ltd., Tokyo, Japan). The AFM operated in tapping mode, employing NSC 15 cantilevers (MikroMasch, Tallinn, Estonia) with a resonant frequency of 325 kHz and a force constant of 40 N/m. Topographic images were captured over an area of 20 × 20 μm at a minimum of three distinct macroscopic locations on the sample surface, with scan rates ranging from 1.5 to 3 Hz, depending on the surface corrugation. The resulting images were analyzed using Jeol WIN SPM 2.0 software (JEOL Ltd., Tokyo, Japan) to determine surface roughness parameters Ra and Rq [15,16].

### 2.4. Vickers Hardness Test

Hardness testing was carried out using a Duramin-40 AC3 instrument (Struers GmbH, Fellbach, Germany). The Vickers hardness number (VHN) test was performed with a load of 2 kgf, a magnification of 2.5×, and an indentation duration of 30 s.

### 2.5. Statistical Analysis

All data were analyzed using statistical software (OriginLab 2019b, Northampton, MA, USA). Means and standard deviations (SDs) were calculated, and correlation tests were applied. The values obtained were subjected to a one-way ANOVA, followed by post hoc Tukey tests. A *p*-value of less than 0.05 was considered statistically significant.

## 3. Results

### 3.1. Dosimetry of Implants After I-131 Irradiation

**Figure 1:**
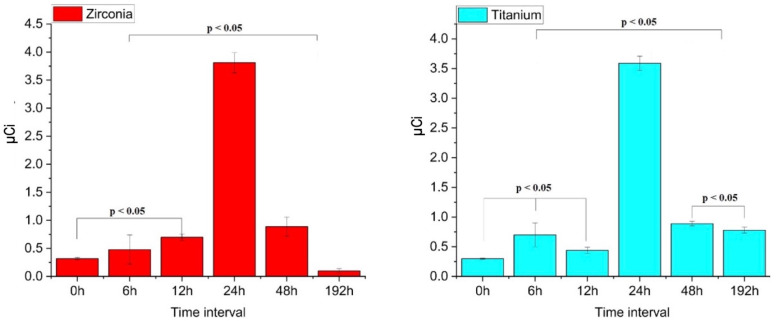
Dosimetric measurements of titanium and zirconia implants.

The comparison between the intervals for Zr and Ti implants showed no statistically significant difference (*p* = 0.96), indicating similar behavior. For the Ti implants, the results across the six time intervals did not show statistical significance for the following comparisons: control and 12 h; 6 and 192 h; 48 and 192 h; and 6 and 48 h. Similarly, for the Zr implants, no statistical significance was observed for the comparisons between the following: control and 6 h; 6 and 12 h; 12 and 48 h; and 48 and 192 h (Figure 1).

### 3.2. Scanning Electron Microscopy Analysis

**Figure 2:**
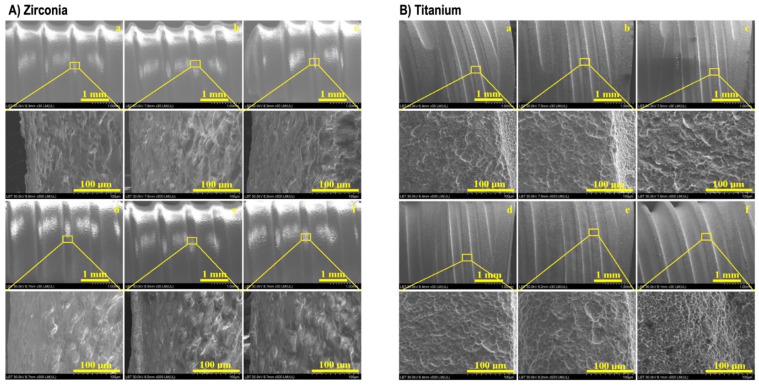
SEM images of the (**A**) zirconia and (**B**) titanium implants exposed to I-131: (**a**) 0 h, (**b**) 6 h, (**c**) 12 h, (**d**) 24 h, (**e**) 48 h, and (**f**) 192 h (macroscopic aspect and microstructural details).

Macrostructural observations revealed that the Zr implants initially exhibited a bright and flawless surface (Figure 2A(a)), with a well-defined thread helix. No significant macrostructural changes were observed within the first 6–12 h of treatment (Figure 2A(b,c)). However, after 24 h of irradiation, the threaded surface began to darken due to minor alterations (Figure 2A(d)), which became more pronounced at 48 and 192 h. Microstructural changes emerged after 24 h, characterized by the formation of small deposits over the sintering necks (Figure 2A(d)). These deposits progressively accumulated over time, further enhancing the embossed appearance of the microstructural details due to debris buildup (Figure 2A(e,f)).

The initial exposure of the Ti implants revealed a porous microstructure (Figure 2B(a)). After 6–12 h of treatment, the microstructure remained well preserved, with the crest margins maintaining their definition and showing no visible alterations (Figure 2B(b,c)). However, by 24 h, early signs of erosion appeared on the pore walls, likely due to interaction with the I-131 solution. This erosive effect became progressively more pronounced over time, leading to significant microstructural changes at 48 h (Figure 2B(e)), including the rounding of crest margins. By 192 h of I-131 exposure, severe surface degradation was evident, with extensive erosion of the pores and noticeable alveolar deterioration (Figure 2B(f)).

### 3.3. Atomic Force Microscopy Analysis

**Figure 3:**
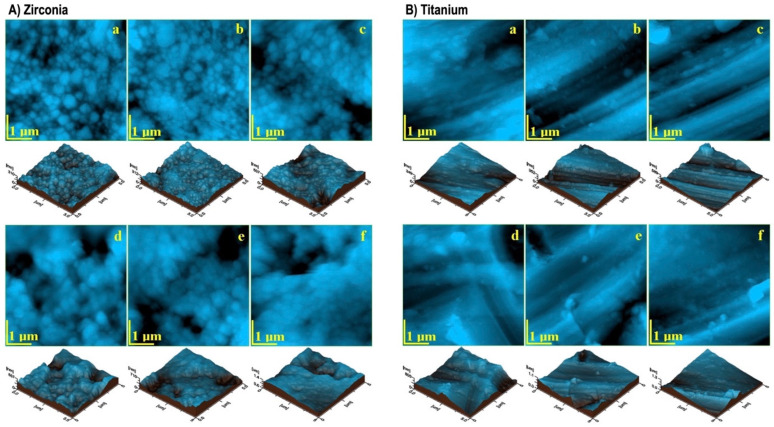
AFM topographic images of the (**A**) zirconia and (**B**) titanium implants exposed to I-131: (**a**) 0 h, (**b**) 6 h, (**c**) 12 h, (**d**) 24 h, (**e**) 48 h, and (**f**) 192 h (macroscopic aspect and microstructural details).

The irradiation effect had minimal impact on the ultra-structural topography of zirconia (Figure 3A(b,c)). The submicron formations remained compact and well organized, maintaining a dense and smooth surface. However, a fine pellicle, likely a result of irradiation, began to form and became more noticeable after 12 h (Figure 3A(c)). After 24 h of I-131 exposure, topographical changes emerged, characterized by smaller crystallites undergoing surface degradation, leading to the formation of rounded depressions approximately 1 µm in diameter (Figure 3A(d)). ZrO_2_ crystallites (100 nm) could detach from the surface, mixing with precipitate salts from the treatment solution to form ultra-structural debris which partially covered larger zirconia grains (400 nm) (Figure 3A(e)). This process gradually roughened the surface, with increasing irregularities observed after 48 h. By 192 h, extensive debris deposition further altered the surface morphology, highlighting a cumulative effect of prolonged irradiation (Figure 3A(f)).

Alterations in the Ti implant surfaces became noticeable after 12 h of exposure (Figure 3B(c)), as the finest ultra-structural grains began to erode, with dislodged material aggregating into debris clusters. These clusters had grown larger and more prominent by 24 h (Figure 3B(d)). Two diffusion flows were observed, partially covered by a foamy debris layer which disrupted the original ultra-structure and contributed to the formation of large cluster deposits. The most significant changes occurred at 48 and 192 h (Figure 3B(e,f)), when pronounced erosive patterns emerged, accompanied by substantial debris accumulation, further altering the surface morphology.

### 3.4. Roughness Analysis

**Figure 4:**
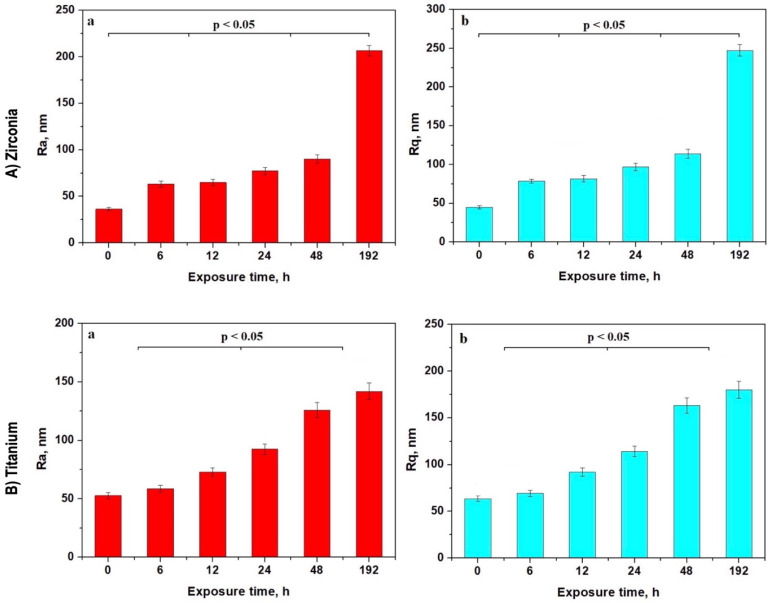
(**A**) Zirconia and (**B**) titanium surface mean roughness variations with the irradiation time: (**a**) Ra and (**b**) Rq.

The roughness variation for Zr implants (Figure 4A) revealed four statistically significant groups (*p* < 0.05). The first group consisted of the initial zirconia surface. The second group included samples exposed for 6 to 12 h, in which a thin pellicle deposit slightly altered the surface. The third group comprised samples treated for 24 and 48 h, during which roughness significantly increased due to the formation of erosion depressions. The fourth group consisted of samples irradiated for 192 h, presenting extensive areas affected by superficial erosion.

The surface roughness of the Ti implants exhibited a gradual increase after 6 h of exposure, forming a statistically significant group compared to the unexposed samples (*p* < 0.05) (Figure 4B). Significant development of the erosion debris clusters was observed after 12 h, further increasing the surface roughness. This cumulative trend resulted in a progressive increase in surface roughness by 24 h of exposure, with samples exposed for 12 and 24 h forming a statistically significant group. This indicated the beginning of the accumulation of alteration faults on the implant surface.

### 3.5. Vickers Hardness Analysis

**Figure 5:**
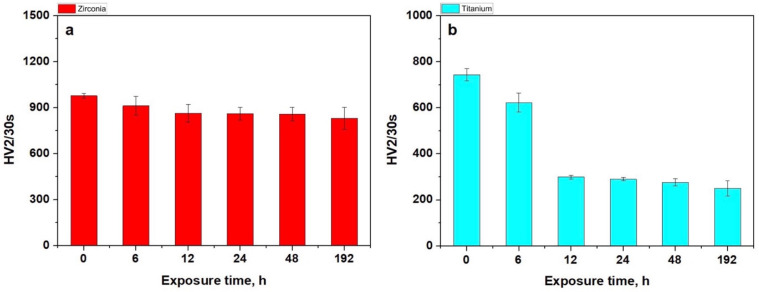
Mean hardness variation: (**a**) zirconia and (**b**) titanium implants.

The surface alterations induced by exposure to the I-131 solution were assessed using SEM and AFM, confirming the formation of erosion debris deposited over the microstructural features. These newly formed deposits were expected to impact surface hardness. Therefore, Vickers HV2 indentation tests were conducted in triplicate for each sample, and the mean values are plotted in Figure 5.

## 4. Discussion

This in vitro study aimed to evaluate the effects of the I-131 solution on Zr and Ti implants. At time 0, the Zi implants showed a measured activity of 0.3 μCi due to the initial adsorption of I-131. Although the implants had been rinsed to remove unbound radioiodine, residual activity remained. By 192 h, activity decreased to 0.1 μCi, likely due to radioactive decay and the desorption of loosely bound radioiodine. The increase in activity within the first 24 h was due to I-131 adsorption on the implant surfaces. The Ti implants, with their porous structure, retained more I-131 than the smoother zirconia implants, leading to higher activity (Figure 1). The unexpected doubling of activity at 192 h for the titanium implants might have been explained by the surface desorption and re-adsorption of I-131 ions. 

The microstructure of the Zr implants was characterized by ZrO_2_ micro-particles sintered into a dense ceramic body. The sintering process promoted diffusion at the contact points between adjacent particles, forming adhesion necks which progressively widened to create the elongated crystallite structure observed at 500× magnification in Figure 2A(a). This specific microstructure was well preserved during the initial stages of treatment, up to 12 h (Figure 2A(b,c)). However, as the exposure time increased, erosion depressions enlarged to 2–3 µm, exhibiting a dendritic appearance with debris deposits predominantly accumulated along their edges. The ultra-structural damages on the Zr surface were significant but confined to the surface layer due to the dense nature of the ceramic material (Figure 3A(f)).

The investigated Ti implants exhibited a smooth root of the thread helix and well-defined crests with a compact structure and correct profile. Structural alterations in Ti began to manifest after 24 h of I-131 treatment, consistent with previous research [17], leading to morphological changes in the pore’s alveoli (Figure 2B(d)).

The initial hardness of the Zr implants aligned with literature findings [18,19]. Over time, the hardness of the Zr implants exhibited a gradual, slight decrease due to the deposition of erosion debris, impacting surface behavior (Figure 5a). This slight reduction was attributed to the high compactness and cohesion of zirconia ceramic, which confined erosive effects to the outermost layers. Conversely, the initial hardness of the unexposed titanium screws corresponded well with data in the literature [20]. However, the Ti implants possessed a porous microstructure that allowed erosive effects to penetrate deeper, corresponding to the depth of the alveoli (Figure 5b). Consequently, the eroded topography expanded, weakening the areas clogged with debris within the porous alveoli, leading to a pronounced, progressive decrease in surface hardness.

In terms of overall success, according to the literature, both implants demonstrate high survival rates: 98.8% for the Ti implants [21] and 95.1% for the Zr implants [22] at a 10-year follow-up. Titanium implants remain the standard material in oral implantology. However, studies indicate that Zr implants may offer a superior soft tissue response, suggesting potential advantages in reducing inflammation and a lower susceptibility to peri-implantitis [23].

In terms of Ti implant survival rates in radiotherapy patients, Schiegnitz et al., in their meta-analysis, reported a high incidence of implant failure among irradiated individuals (OR 1.97; CI [1.63, 2.37]; *p* < 0.00001) [24]. Camolesi et al., in their meta-analysis, observed a 5-year follow-up survival rate of 93.13% (95% CI: 87.20–99.06; *p* < 0.001) for irradiated patients compared to 98.52% (95% CI: 97.56–99.48, *p* < 0.001) for non-irradiated patients [25]. Shokouhi and Cerajewska noted a higher implant survival rate in the mandible compared to the maxilla (*p* = 0.04) [26]. The occurrence of osteoradionecrosis (ORN) in conjunction with dental implant placement has also been recognized, with an incidence rate of 3% [27]. It is generally recommended to carefully assess replacement options for gaps, select cases thoughtfully with comprehensive medical and dental optimization, and involve a restorative specialist before initiating radiotherapy to optimize implant site selection [27].

Regarding the use of dental implants in patients undergoing I-131 therapy, there is limited research available. Focal oral uptake observed in diagnostic I-131 scans often corresponds to high-density dental materials detected on SPECT/CT scans. Savas et al. suggested that persistent focal I-131 accumulation in the oral cavity results from an electrostatic attraction between negatively charged iodide ions in the saliva and positively charged metal ions in dental materials [28]. In a prior review, our research team highlighted that oral complications can disrupt protocols for radioiodine I-131 therapy, potentially requiring adjustments in dosage, modifications to the treatment regimen, or discontinuation of therapy. Dental professionals play a crucial role in preventing and managing oral side effects associated with radioiodine treatment. Recognizing the importance of maintaining salivary gland health is paramount for overall periodontal health [6]. Regarding this aspect, our main contribution with this paper was to determine, in vitro, the stability of zirconia implants when irradiated with I-131. Consequently, from a clinical point of view, titanium implants still remain the first choice of material. Future clinical trials might be useful to assess the benefits of Zr implants in irradiated patients.

## 5. Conclusions

Our in vitro analysis revealed that the microstructure of both Zr and Ti implants begins to change 24 h after I-131 therapy. By 192 h of I-131 exposure, both types of implants exhibit alterations on their crest surfaces. Ti implants show larger erosive patterns accompanied by significant debris deposits.

## Data Availability

The data presented in this study are available upon request from the corresponding authors.
